# Supplementary material for: Complex‐centric proteome profiling by SEC‐SWATH‐MS
Source: Mol Syst Biol. 2019 Jan 14;15(1):e8438. doi: 10.15252/msb.20188438 (PMC6346213; doi:10.15252/msb.20188438)
Supplement: Supplementary file 8 — Dataset EV7 [file MSB-15-e8438-s008.zip › feature_plots_string/Q9NYA1.pdf]

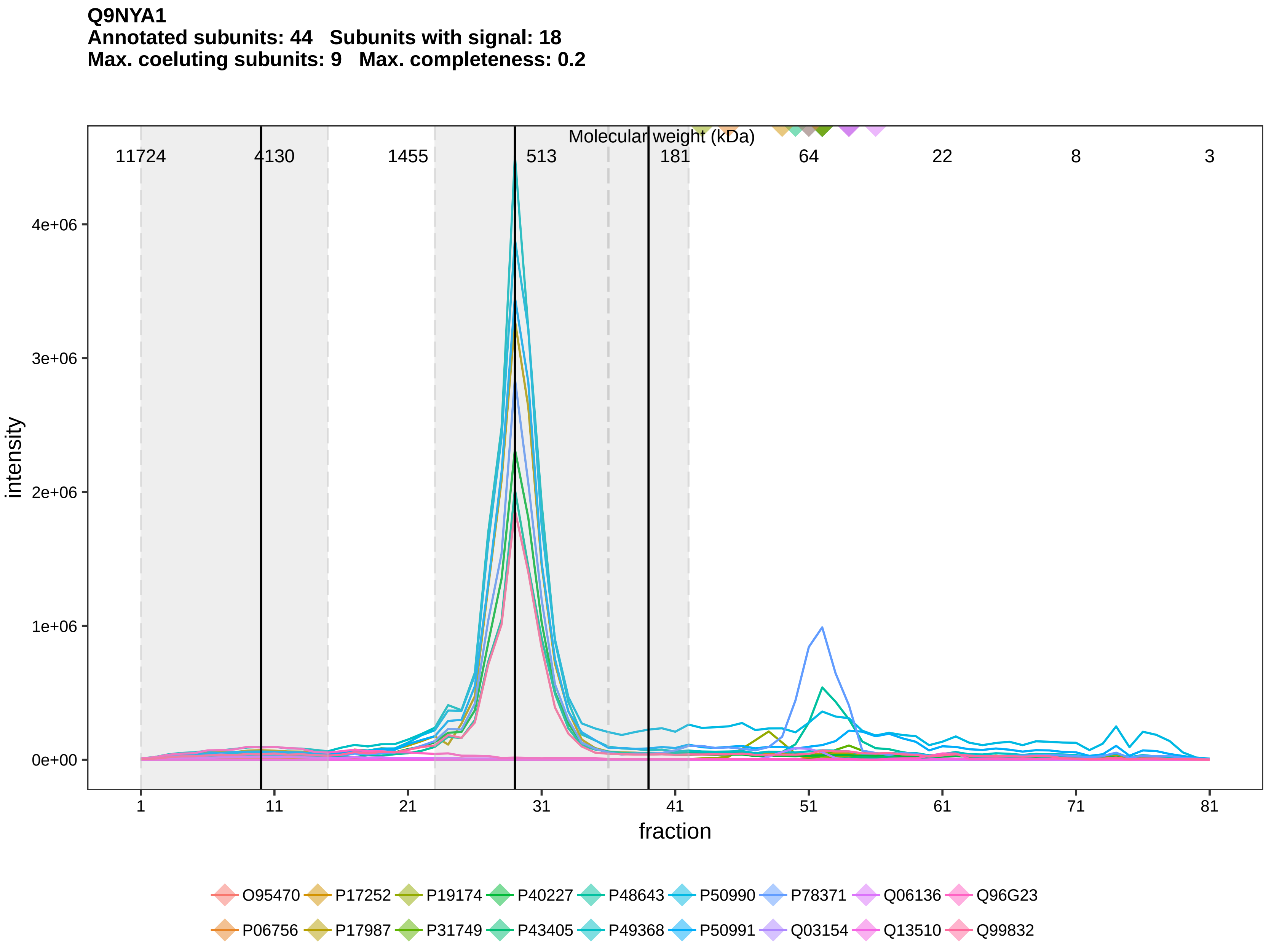

Q9NYA1
Annotated subunits: 44 Subunits with signal: 18
Max. coeluting subunits: 9 Max. completeness: 0.2
Molecular weight (kDa)
11724
4130
1455
513
181
64
22
8
3
intensity
4e+06
3e+06
2e+06
1e+06
0e+00
fraction
1
11
21
31
41
51
61
71
81
O95470
P17252
P19174
P40227
P48643
P50990
P78371
Q06136
Q96G23
P06756
P17987
P31749
P43405
P49368
P50991
Q03154
Q13510
Q99832
